# Medical Society of Tennessee

**Published:** 1843-07

**Authors:** 


### MEDICAL SOCIETY OF TENNESSEE.

The meeting of this Society was held at Nashville on the first Monday in May, as usual, and was attended by about the usual number of members. The cases reported were generally interesting, and a number of them will be published in our Journal. Dr. Richardson, of Rutherford county, delivered a sensible, practical address, as orator for the occasion. The Society resolved on founding a Cabinet of Pathological Anatomy, in Nashville, and solicit specimens of the healthy as well as of morbid structure from all its members, and from physicians generally. Y.

